# Machine Learning Methods to Detect Voltage Glitch Attacks on IoT/IIoT Infrastructures

**DOI:** 10.1155/2022/6044071

**Published:** 2022-04-26

**Authors:** Wei Jiang

**Affiliations:** Zhengzhou College of Finance and Economics, Zhengzhou 450000, China

## Abstract

A majority of modern IoT/IIoT digital systems rely on cryptographic implementations to provide satisfactory levels of security. Hardware attacks such as side-channel analysis attacks or fault injection attacks can significantly degrade and even eliminate the desired level of security of the infrastructure in question. One of the most dangerous attacks of this type is voltage glitch attacks (VGAs), which can change the intended behavior of a system. By effectively manipulating the voltage at a specific time, an error can be injected that can change the intentional conduct and bypass system security features or even extract confidential information such as encryption keys by analyzing incorrect outputs of the firmware. This study proposes an innovative VGAs detection system based on advanced machine learning. Specifically, an innovative semisupervised learning methodology is used that utilizes a hybrid combination of algorithms. Specifically, a heuristic clustering method is used based on a linear fragmentation of group classes. In contrast, the ELM methodology is used as an algorithm for retrieving hidden variables through convex optimization.

## 1. Introduction

The Internet of Things (IIoT) is a network of networked sensors, instruments, and other devices that, when combined with industrial applications such as production and energy management, provide a complex system of services that allows for higher-level automation [1, 2]. Data collection, exchange, and analysis are substantially facilitated by this connectedness, which greatly aids performance improvement throughout the value chain. Physical systems such as sensors, actuators, control systems, security mechanisms, and other IIoT systems are frequently combined as a multi-layered digital technology architecture, where physical networking media (wired and wireless) protocols that collect and transfer information to the upper and lower layers of the communications layer are mentioned at the hardware level, while at the network level, physical networking media (wired and wireless) and protocols that obtain and send data to the upper and lower layers of the communications layer are mentioned [3].

Cyber-physical platforms [2, 4] are super-grid interactive computer and communication technologies that use feedback loops to monitor, coordinate, and control physical elements. Physical processes impact IoT computations and vice versa [5]. This solution combines the dynamics of physical processes with those of software and networking, resulting in abstract technical analysis and design models for a unified whole that is more akin to the intersection than the merger of the physical and digital worlds. Cyber-physical systems are a new generation of sophisticated capabilities that use information technology, communications, precise control, coordination, and autonomy to achieve physical association with the digital environment [3]. Understanding the standard components, the dynamics of information systems, hardware, software, networks, and the physical processes that model a scenario, as well as the relationships between them, is required for their design [6].

Industry 4.0 [3] defines cyber-physical workflow as an optimal combination of equipment and items, encompassing production facilities, storage mechanisms, enterprise resource planning, manufacturing execution system, outbound logistics, and service provisioning [7, 8]. They are integrated systems encompassing the production cycle and storing and evaluating the generated data for industrial process modeling and analysis. Intelligent machines communicate via machine-to-machine (M2M) communication, performing controls on both sides and making decentralized judgments. The communication network and other intermediary elements are the interfaces that interact with the normal interfaces of the physical with the digital world [9–11].

As it is widely understood, the design of low power circuits is a critical operational factor of Industry 4.0, where devices such as interconnected sensors, actuators, and digital-analog signal converters are actively integrated into the IoT as autonomous mechanisms of the production process [3, 10, 12]. In low power combinational circuits, the dynamic power supply can receive a signal transition either as a functional or glitch. Before it reaches a steady state, a signal can go through many static changes called glitches. As glitches dissipate 20–70% of the total power consumption, they play a vital role in their operation, so it is necessary to control them thoroughly for the smooth operation of low power circuits [13].

The presence of hardware attacks such as side-channel analysis attacks and fault injection attacks can significantly degrade and even eliminate the desired level of security of low power circuits included in Industry 4.0 [5]. Such physical attacks are numerous and can be classified into two main categories as follows [13–15]:

1. Invasive/noninvasive: invasive attacks necessitate interfering with the chip shell to gain direct access to the chip's interior components. Connecting a cable to a data bus to access data transfers is a good illustration of this. Noninvasive attacks, on the other hand, rely solely on externally available data (sometimes inadvertently emitted) such as operational time and power consumption.
2. Active/passive: active attacks aim to stop equipment from functioning properly. Error-induced assaults, for example, will attempt to introduce computational errors. Passive assaults, on the other hand, will just watch the behavior of the devices throughout processing without interfering with it.

In general, the above is also referred to as implementation attacks. They include any effort that is dependent on information derived from an electronic system's implementation rather than flaws in the implemented algorithm itself (e.g., cryptanalysis and software implementation mistakes) [16, 17]. Timing information, power consumption, electromagnetic leakage, and even sound can all be used as supplementary data sources. Side-channel attacks, fault attacks, optical fault injection, electromagnetic fault injection, clock/voltage glitch, and other examples of this sort of attack vary depending on the medium utilized. [13, 15].

The most dangerous and difficult-to-detect type of attack is the VGAs [14, 15]. It is achieved at a physical level and interferes with the operation of the material by applying physical disturbances or changing environmental conditions, for example, using heavy-ion radiation and magnetic or electronic interference. These disturbances can cause the supply voltage to fluctuate (supply disturbances), introduce laser memory errors, or modify the input/output value of the circuit. Error input based on this type of attack may also include the addition of specially designed hardware to the system under evaluation, which allows the introduction of specific kinds of errors and the monitoring of costs to examine the effects of errors on system operation [13, 16, 18]. Depending on their mistake and location, VGAs fall into two categories as follows [13, 15, 19]:

1. Contact fault input: direct physical contact with the target system, causing voltage or current disturbances in the target chip.
2. Noncontact hardware error input: there is no direct physical contact with the target system. Instead, an external source produces a natural phenomenon like heavy-ion radiation or electromagnetic interference that causes the target chip to malfunction.

Dealing with the highly complex and undetectable attacks of hardware-related VGAs is an open problem in the research community, both in hardware development and digital security, as reflected in the international literature.

## 2. Literature Review

The massive increase in data flow across IoT sensors and, more importantly, in IIoT communication protocols has raised security concerns, emphasizing the significance of reliable approaches for promptly and accurately identifying threats. Security professionals and researchers rely on automated methods aided by deep learning to improve the efficacy of unwanted behavior detection, which is gaining popularity in the corporate world.

Sengupta [6] conducted a comprehensive review of IoT security concerns and countermeasures, with a focus on IIoT, and classified attacks based on the vulnerability object. This classification would make it easier for scholars to figure out which attacks are relevant to their particular field of study. Following that, each attack is mapped to one or more layers of the generic IoT/IIoT architecture, followed by a discussion of the available defenses. Researchers would also have a better understanding of the major security research concerns and their solutions in the field of IoT/IIoT by using a complete taxonomy. Finally, they present a case study on two critical industrial IoT applications.

Barenghi et al. [15] concentrated on fault injection attacks that did not have specific hardware or capabilities. They presented a detailed overview of these cryptographic device attacks and the solutions that have been devised to combat them. They compiled a list of attacks for the most important and widely used ciphers, stating which ones have been successfully implemented. They divided fault injection attacks into two categories as follows: low cost and high cost. They went over the protections, including intrusion detection and fault diagnosis, before examining the connection between fault injection and power analysis threats.

Vosoughi and Köse [16] advocated using the on-chip voltage regulator's existing resources as a countermeasure against VGA to improve their durability. They compared the number of phases in the multi-phase voltage regulator (MPVR) to the number of phases in the VGA. On a substitution box (S box) of an AES, they tested the efficiency of the proposed countermeasure. When compared to the unprotected S-box of an AES device, the faults induced by the VGA on the cryptographic circuit were reduced by 5.45% with a single-phase on-chip VR and by 91.82% with an MPVR with 32 phases, demonstrating the efficacy of their technique.

Bozzato et al. [13] introduced the voltage fault injection (V-FI) approach, which uses off-the-shelf and low-cost equipment to generate completely arbitrary voltage glitch waveforms. They looked into the possibility of automatically and unsupervised detecting a valid set of attack parameters, including the glitch waveform. The results revealed an increase in firmware extraction speed and a significant reduction in the number of injected bugs needed to accomplish the attack. They also demonstrated previously unknown firmware extraction attacks on six microcontrollers from three major brands, which targeted the bootloader interface and extracted the firmware from the internal protected flash memory. The most difficult attacks shown exploit numerous vulnerabilities and inject over one million flaws, relying primarily on the newly proposed technique's performance and repetition. They demonstrated that an attacker could employ voltage fault injection to defeat the safeguards supplied by the microcontrollers under test, even with low resources.

Software attacks targeting hardware vulnerabilities was a term used by Polychronou et al. [20] to describe a specific class of malicious attack vectors targeting IoT/IIoT devices (SATHV). These techniques are aimed at both the hardware flaws in system microarchitecture and the side-channel leakages they cause in the system, and they do not require physical access to the device. They also recommended security measures that might be used to prevent sensitive data from being extracted, malicious implant code from being implanted, and privileged code from being accessed. They attempted to educate designers on the negative consequences of attacks and detection measures outlined in the literature. They offered two tables based on the criteria that listed and classified the side effects and detection mechanisms. They believe that IoT/IIoT systems require more robust security solutions because, in addition to the ease of attacks, defenders do not realize which attack routes will be employed in advance, thus they must design and optimize numerous detection techniques at the same time.

For the first time in the literature, our work proposes a heuristic semisupervised learning method, which uses a simplified methodology for linear segmentation of groups classes. Using an extremely simple and fast ELM [21] recovers the hidden variables that lead to the problem's solution. It is important to note that most of the solutions proposed are well-defined techniques that include microprocessor-type solutions, special hardware, countermeasure technologies, etc., which are very difficult to impossible to be a widely accepted solution.

## 3. Materials and Methods

To detect VGAs, we first model the problem of clustering N data into P classes and the set of P classes. Every data *x*_*i*_ with *i* ∈ *N* = {1, 2, ..., *N*} belongs to the space *R*^1×D^. We define table X ∈ R^N×D^ with lines *x*_*i*_. Each sample *x*_*i*_ belongs to a class of P. We define the variable *z*_*i*_ with *i* ∈ *N*, which belongs to the space {0, 1}^1× P^ with *z*_*i*_ 1P = 1, that is, a binary variable of dimension *P* that takes the value one only at a position *p* if and only if the data belong to the class *p*. Similar to *x*_*i*_, we define the variable Z ∈ R^N×P^ with lines *z*_*i*_ and the set of index tables *Z*_*N,P*_ = {Z ∈ {0, 1}^N×P^|*Z*·1_*P*_ = 1_*N*_}. This variable is a latent variable as we do not have access to the ground truth of the data. The purpose is to retrieve the values of the hidden variable and at the same time to train the ELM classifier h: *R*^*D*^ ⟶ *Z*_1,*P*_, which will accept a characteristic vector data of dimension *D* as input and will return the index vector of the class to which the data belong. We can choose the classifier as follows [10, 11, 13, 21]:

(1) $$\begin{array}{c} f\left(x\right)=\left(f_{1}\left(x\right),f_{2}\left(x\right),\ldots ,f_{P}\left(x\right)\right),\\ h\left(x\right)=\left(h_{1}\left(x\right),h_{2}\left(x\right),\ldots ,h_{P}\left(x\right)\right),\\ h_{j}\left(x\right)=\left\{\begin{array}{cc} 1, & j=\underset{i}{\text{arg max}}f_{i}\left(x\right),\\ 0, & \text{elsewhere,} \end{array}\right. \end{array}$$

where *f*: *R*^D^ ⟶ *R*^P^.

Extending the equation to the problem of unknown classes, the objective function is also minimized for *z*_*i*_ as follows [22–24]:

(2) $$\begin{array}{c} \underset{Z,f}{\text{min}}\frac{1}{N}\underset{i\in N}{\sum }\ell \left(z_{i},f\left(x_{i}\right)\right)+\lambda \Omega \left(f\right). \end{array}$$

Let us consider that the data are displayed in a space where the classes are linearly separable (the partition surfaces for each pair of classes are superficial). The function *f* can take the following form:

(3) $$\begin{array}{c} f\left(x\right)=xw+b,\;w\in {\mathbb{R}}^{D\times P}\;,b\in {\mathbb{R}}^{1\times P}. \end{array}$$

Finally, if we define the function as the square error and the normalization term as the *L*_2_ norm of *w*, then the problem takes the following form:

(4) $$\begin{array}{c} \underset{Z,w,b}{\text{min}}\frac{1}{2N}Z-Xw-1_{N}b_{F}^{2}+\frac{\lambda }{2}\text{Tr}\left(w^{T}w\right). \end{array}$$

Holding the *Z* as constant, we can find the minimum value of the function on *w* and b in closed form. To find the coefficients in this way, the ELM methodology is used as an algorithm for retrieving hidden variables through the solution of a convex program [21, 25].

ELMs are feedforward single hidden-layer feedforward neural networks (SLFNs). Given N random discrete observations {(*x*_*i*_, *t*_*i*_)} for *i* = 1 as Ν, where *x*_*i*_ ∈ *R* n with *x*_*i*_  = [*x*_*i*1_, *x*_*i*2_,…, *x*_*in*_] *T* and *t*_*i*_ ∈ *R* m with *t*_*i*_  = [*t*_*i*1_, *t*_*i*2_,…, *t*_*im*_] *T*, an ELM with hidden nodes (neurons) K and activation function *g*(*x*) is mathematically modeled with the following formula [21, 22]:

(5) $$\begin{array}{c} f\left(x_{j};w,b,\beta \right)=\overset{K}{\underset{i=1}{\sum }}\beta_{i}*g\left(w_{i}*x_{j}+b_{i}\right)=o_{j},j=1,2,\ldots ,N, \end{array}$$

where the variable *w*_*i*_ = [*w*_*i*1_, *w*_*i*2_,…, *w*_*in*_] *T* is the vector of weights that connects the node *i* of the hidden plane with the nodes of the input plane, *β*_*i*_  = [*β*_*i*1_, *β*_*i*2_,…, *β*_*im*_] *T* is the vector of weights that connects the node *i* of the hidden level with the nodes of the output layer, and *b*_*i*_ is the threshold of the hidden node *i*. A typical SLFN with hidden nodes K and activation function g(x) can approach N random observations with zero mean error value [21]:

(6) $$\begin{array}{c} \sum_{j=1}^{K}\left\|o_{j}-t_{j}\right\|=0. \end{array}$$

Therefore, there are *β*_*i*_, *b*_*i*_, and *w*_*i*_ such that

(7) $$\begin{array}{c} f\left(x_{j};w,b,\beta \right)=\overset{K}{\underset{i=1}{\sum }}\beta_{i}*g\left(w_{i}*x_{j}+b_{i}\right)\\ =t_{j},\;j=1,2,\ldots ,N. \end{array}$$

For a given SLFN, there are *N* such equations (as many nodes of the hidden layer) that can be written as follows [26]:

(8) $$\begin{array}{c} H\beta =T, \end{array}$$

where the array **H** is the output of the hidden layer.

(9) $$\begin{array}{c} H_{NxK}=\left[\begin{array}{ccc} g\left(w_{1}*x_{1}+b_{1}\right) & \cdots & g\left(w_{K}*x_{1}+b_{K}\right)\\ \vdots & \ddots & \vdots \\ g\left(w_{1}*x_{N}+b_{1}\right) & \cdots & g\left(w_{K}*x_{N}+b_{K}\right) \end{array}\right]. \end{array}$$

Table *β* symbolizes the table of output weights:

(10) $$\begin{array}{c} \beta =\left[\begin{array}{c} \beta_{1}\\ \beta_{2}\\ \cdots \\ \beta_{K} \end{array}\right]. \end{array}$$

And, **T** is the table of the desired output values:

(11) $$\begin{array}{c} T=\left[\begin{array}{c} t_{1}\\ t_{2}\\ \cdots \\ t_{m} \end{array}\right]. \end{array}$$

The training process aims to find values for the variables *w*_*i*_, *b*_*i*_, and *β*_*i*_ for *i*  = 1,2,…, *K* for which it applies [21, 21]:

(12) $$\begin{array}{c} \left\|H*\hat{\beta }-T\right\|=\underset{w_{i},b_{i},\beta_{L}}{\text{min}}\left\|H*\beta -T\right\|, \end{array}$$

which corresponds to minimizing the cost function.

(13) $$\begin{array}{c} E=\sum_{j=1}^{N}{\left(\sum_{i=1}^{K}\beta_{i}*g\left(w_{i}*x_{j}+b_{i}\right)-t_{j}\right)}^{2}. \end{array}$$

According to the backpropagation algorithm, a gradient descent algorithm is used to find the value:

(14) $$\begin{array}{c} \underset{w_{i},b_{i},\beta_{t}}{\text{min}}\left\|H*\beta -T\right\|. \end{array}$$

In the minimization process, the vector **W**, which is the sum of the weights (*w*_*i*_, *b*_*i*_) and the biases (*β*_*i*_), is adjusted iteratively according to the following relation [26]:

(15) $$\begin{array}{c} W_{k}=W_{k-1}-n\frac{\partial E\left(W\right)}{\partial W}, \end{array}$$

where *n* is the learning rate of the neural network. We used an easy-to-use, simple, and fast ELM as an algorithm for retrieving hidden variables in problem-solving. This heuristic methodology performs a linear fragmentation of class groups semiautomatically [21].

## 4. Experiments

To implement the scenario of the use of the proposed algorithm, the exact ways and the main factors that contribute to the energy consumption in the combined microcircuit circuits were studied. While the inputs of a combination circuit are excited by flip-flops, the internal gates of the circuit may need several shifts until they reach a steady state. These extra transitions are called glitches [27, 28]. Although not anticipated by designers, they are not necessarily design errors in terms of logical behavior. Still, they are a big problem in terms of digital security due to the fact that extra transitions consume energy. This form of energy is also known as glitch power and is quite tricky to calculate accurately [27, 29]. All experiments were conducted in the Google Colab no-GPU environment.

The percentage of the total energy that can come from glitches, which can be legitimately based on the circuit design and illegal due to VGAs, is quite large and difficult to calculate accurately. Since a percentage of the total power consumption diffuses into a circuit due to glitches, the tools for estimating the total power must be accurate in the presence of this phenomenon. This can be done electrically but only for medium-sized circuits. On the other hand, reasonable accuracy has not yet been achieved in detail. A distinctive feature of static circuits is that the total power consumption is mainly caused by signal switching. Therefore, logic gateway-level simulation algorithms calculate the average power dissipated by monitoring the activity (e.g., number of transitions) of a gateway output using the following relation [13, 16, 30]:

(16) $$\begin{array}{c} P_{\text{avg}}=f\frac{V_{D\;D}^{2}}{2}\sum_{i}^{n}C_{L_{i}}a_{i}, \end{array}$$

where ƒ is the clock frequency and *n* is the number of gates. At the same time, *C*_*Li*_ and *a*_*i*_ are the output capacity and the number of gate transitions of gate *i* during the period under consideration, respectively. It is important to note that the above relation does not consider the power consumed by the internal capacitors and by the short-circuit currents. The total power consumption of a circuit consists mainly of dynamic power consumption and static power consumption, which include other components respectively, as shown in the following equation [17, 31, 32]:

(17) $$\begin{array}{c} \bar{P}=f_{\text{clk}}\int_{0}^{T_{\text{clk}}}Vdd^{*}I_{\text{supply}}\text{d}t. \end{array}$$

The input signals of a gateway are varied in such a way as to produce a value at the output of the gateway. However, depending on the time at which the signal changes take place, there is a possibility that an additional output value will be generated, resulting in a static glitch [27, 29, 30].

In the present work, a simulation was created that deals with the analysis and study of glitches made in the logical NAND 2 input gate designed at 1.2 *μ*m and with a supply voltage of 1.1 V. There are two ways that a glitch can appear on this portal. The first is to create the glitch in this gate, which is done by the appearance of two transitions at its entrances with very close arrival times and the logical behavior of the gate to lead to it. The second is by propagation through the gate, wherein in this case, a glitch reaches the entrance of a gate and causes a similar situation at the exit node. Creating a glitch on a node spread to the following logical levels until logical or electrical masking can neutralize it [13, 16, 31].

**Figure 1:**
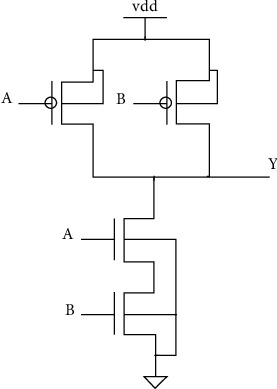
NAND gate (2 inputs).

The 2-input NAND gateway and its schematic simulation to collect glitches used to evaluate the proposed system are shown in Figure 1.

To create a glitch, we need to perform the transition CD = 01 ⟶ 10 and the transition CD = 10 ⟶ 01. We need two transitions of the input signals of the NAND 2 gate from 0 ⟶ 1 and 1 ⟶ 0. Creating a glitch at a node in the circuit begins to spread to the following logical levels until logical or electrical masking can neutralize it. More specifically, the glitches study area has two boundaries [29, 31]:

1. The start time of the transition of one signal is equal to the end time of the transition of the other signal.
2. The start time of the transition of the other signal should not exceed the end time of the transition of the first signal, that is, it should always be *t*_1_ < *t*_2_.

Great attention was paid to this study, so that the analysis is done each time before the procedure begins to avoid the breakdown of areas where glitches cannot occur. A total of 8,890 glitches were generated randomly distributed over a 12-hour time horizon.

Table 1 lists the success rates achieved by the proposed semisupervised algorithm. The values were calculated as the average of the metrics for each time slot, in which the glitches were randomly distributed.

The results are considered satisfactory given the complexity of the problem and the nonuniform classes that indicate the glitches detection problem. In general, the finding is that the proposed system can reliably evaluate and categorize the current anomalies associated with VGAs.

## 5. Discussion and Conclusions

Hardware attacks such as VGAs are among the most important modern attacks on IoT/IIoT devices [20]. Features such as the predicted behavior of the device can be changed, or even secret information such as encryption keys can be changed intercepted [33]. Given the growing complexity, ever-changing distributed industrial environment combined with the weakness of traditional systems, which in most cases fails to adapt to modern challenges, it is necessary to use alternative and more effective methods to protect industrial infrastructures [4, 7].

This study proposes an innovative VGAs detection system based on advanced machine learning. Specifically, an innovative semisupervised learning methodology is used, which utilizes a hybrid combination of algorithms [34]. It is an innovative heuristic nonaccelerated learning method for fragmenting VGAs problem-class groups. At the same time, an ELM is used as an algorithm to retrieve hidden variables for optimal problem-solving. The proposed methodology has serious advantages over other types of learning. Their main advantage, and the reason that makes it an ideal method for predicting short-term trend shifts, is to avoid using the time-consuming, repetitive backpropagation algorithm [35]. The proposed system uses unsupervised learning to determine the unknown distribution of data. At the same time, ELM is limited to a multiplication of tables, which reduces by almost 75% the time required to complete the classification. Also, avoiding the use of retrospective techniques such as backpropagation contributes to the nonappearance of local minima during the model's training, which affects the model's accuracy.

The evaluation of the system was carried out in an innovative data set created based on a highly complex and original scenario related to the operation of IoT/IIoT [36]. The results obtained are very encouraging and reflect the usefulness and effectiveness of machine learning systems in solving complex problems.

Future extensions of this research work should first focus on optimizing the model's hyperparameters to improve the performance and generalization it can achieve significantly. It is also imperative to make a thorough comparison between classical and modern machine learning architectures to understand the predictive power of the proposed method. Finally, self-determination methods should be explored to make the system autonomous.

## Figures and Tables

**Table 1:** Performance of the proposed method.

Time slots	F-1 score (average)	Precision (average)	Recall (average)	Accuracy (average)
T1	81.00	80.90	80.90	80.90
T2	83.40	84.00	84.00	83.80
T3	88.80	88.90	88.90	88.80
T4	82.60	82.90	82.80	82.70
T5	89.90	88.90	89.00	89.00
T6	86.80	86.90	87.00	86.90
T7	90.20	90.30	90.30	90.30
T8	88.00	88.20	88.20	88.00
T9	89.10	89.10	89.00	89.20
T10	90.70	90.70	90.70	90.70
T11	83.90	83.90	83.80	83.90
T12	89.70	89.60	89.60	89.80
Average	87.00	87.00	87.00	87.00

## Data Availability

Data are available on reasonable request to the corresponding author.
